# Improvement of Water Quality at Dongbin Harbor with Construction of an Inland Canal, Korea

**DOI:** 10.1155/2014/721395

**Published:** 2014-06-05

**Authors:** Yong-Sik Cho

**Affiliations:** Department of Civil and Environmental Engineering, Hanyang University, 222 Wangsimni-ro, Seongdong-gu, Seoul 133-791, Republic of Korea

## Abstract

The behaviors of the water body of Dongbin Harbor located at Pohang City, Gyongpook Province, in Korea were numerically simulated in this study. A canal was planned to connect the harbor and the Hyeongsan River to improve water quality inside the harbor. The current system was first simulated by using a commercial program RMA2, with respect to both tidal currents and river flow. The progress inside the harbor from a supply of fresh water from the Hyeongsan River was then predicted by using RMA4. Both the present and future conditions (before and after construction of an inland canal) were taken into consideration in numerical simulations. It is concluded that the water quality inside the harbor can be improved considerably after construction of the canal.

## 1. Introduction

**Figure 1:**
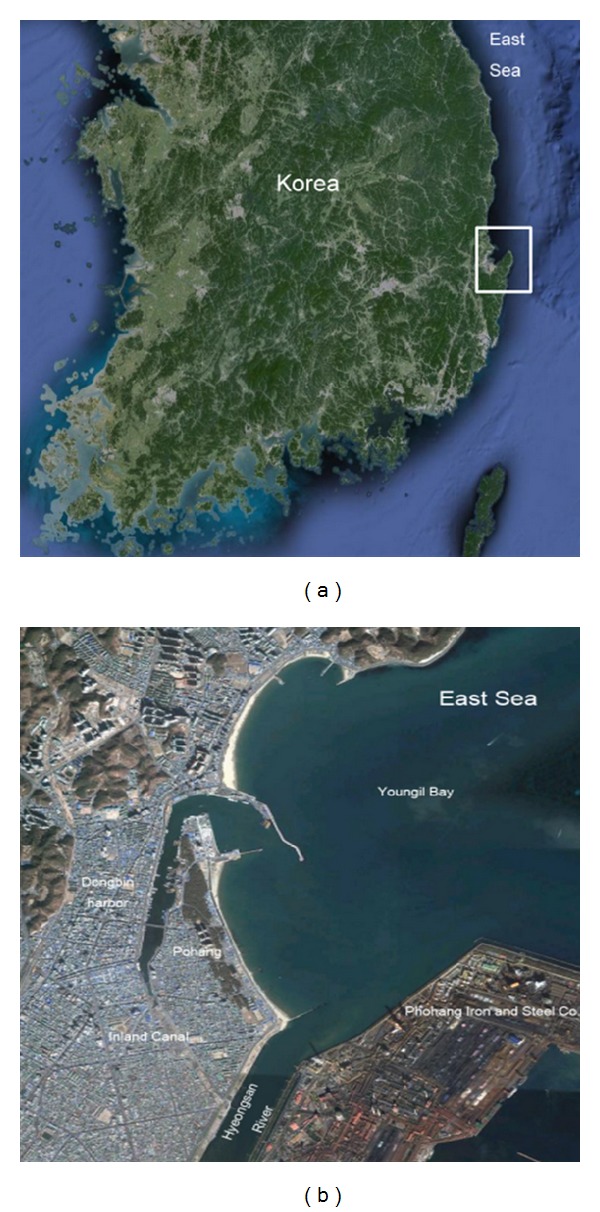
The location of Dougbin Harbor and schematic layout of Dongbin Harbor and the Hyeongsan River.

The water quality of Dongbin Harbor in Pohang City, located at the southeastern area of the Korean Peninsula, is getting worse due to the influx of sewage and wastewater from the surrounding coastal communities. Furthermore, water flow in the harbor is nearly stagnant, because Dongbin Harbor is isolated from the coastal area and is supplied by tidal currents only through Youngil Bay (see Figure 1). Figure 1 shows a general view of Pohang City. The city is famous as the hometown of the Pohang Iron and Steel Company Ltd.

Dongbin Harbor has been transformed into a deep gulf because the stream flow of the Hyeongsan River was stopped by the construction of POSCO (Pohang Iron and Steel Company, Ltd) during the 1970s. The Harbor is surrounded by Youngil Bay and tidal waves in that area range up to 0.22 m, so the limited flow of seawater means the harbor is almost stagnant [1]. Furthermore, a great quantity of wastewater from local communities flows to sea due to rapid industrialization, without any waste water treatment [2]. In an attempt to improve the water quality of Dongbin Harbor, Pohang City has annually dredged the harbor bottom [3]. However, water quality has remained almost unchanged for a long time.

Therefore, the Pohang City has decided to connect the Dongbin Harbor and the Hyeongsan River by constructing an inland canal as shown in Figure 1. Fresh water supplied from the river may improve water quality inside the harbor. In this study, a commercial computer model was used to predict the water quality of the harbor.

RMA2 is a two-dimensional, depth-integrated, free-surface, finite element program for solving hydrodynamic problems. RMA2 was originally introduced in [4]. Many modifications to the original code have been made by a number of researchers [5, 6]. RMA4, a companion model to RMA2, is designed to simulate the depth-integrated advection-diffusion process in an aquatic environment [7, 8]. The model is useful for evaluation of the basic processes and for defining the effectiveness of remedial measures. For most of the applications, the model employs the depth-integrated hydrodynamics from RMA2.

In the following sections, the numerical model is briefly introduced. First, the governing equations and relevant coefficients are described followed by the numerical analysis. The model is first applied to a real field test to validate the applicability. The computational area, boundary conditions, and related coefficients are also introduced. The main focus is predicting the change in COD (chemical oxygen demand) concentration before and after the construction of an inland canal. Numerical results are then shown and discussed. Finally, the concluding remarks are presented.

## 2. Numerical Model

The commercial computer program RMA2 solves the depth-integrated equations of conservation of fluid mass and momentum in two horizontal directions. For completeness, the governing equations consisted of continuity and momentum equations are given by

(1) ∂h∂t+h(∂u∂x+∂v∂y)+u∂h∂x+v∂h∂y=0,h∂u∂t+hu∂u∂x+hv∂u∂y−hρ[Exx∂2u∂x2+Exy∂2u∂y2]  +gh[∂a∂x+∂h∂x]+gun2h1/3u2+v2  −ζVa2cos⁡ψ−2hvωsinΦ=0,h∂v∂t+hu∂v∂x+hv∂v∂y−hρ[Eyx∂2v∂x2+Eyy∂2v∂y2]  +gh[∂a∂x+∂h∂x]+gvn2h1/3u2+v2  −ζVa2sinψ−2huωsinΦ=0,

where *h* is a total water depth (m), *u* and *v* are velocity components in the *x*-, *y*-axis directions (m/sec), *t* is time (sec), *ρ* is the fluid density (998.46 kg/m^3^), *g* is the gravitational acceleration (m/sec^2^), *E* is the eddy viscosity coefficient (Pa-sec), *a* is the channel bottom elevation (m), *n* is Manning's roughness coefficient, *ζ* is an empirical wind shear coefficient, *V*_*a*_ is the wind speed (m/sec), *ψ* is the wind direction, *ω* is the earth's angular rotation rate (sec^−1^), and Φ is the local latitude. Equation (1) is solved by the finite element method by using the Galerkin method of weighted residuals. The companion computer program RMA4 solves a depth-integrated equation of the transport and mixing processes. The depth-integrated transport equation is given as follows:

(2) h(∂c∂t+u∂c∂x+v∂c∂y−∂∂xDx∂c∂x−∂∂yDy∂c∂y  − σ+kc+R(c)h)=0,

where *c* is the concentration of a pollutant for a given constituent (kg/L), *D*_*x*_ and *D*_*y*_ are turbulent mixing (dispersion) coefficients (m^2^/sec), *k* is the first order decay of the pollutant (day^−1^), *σ* is the source or sink of a constituent (kg/L/sec), and *R*(*c*) is the rainfall and evaporation rate (cm/hr). The transport equation is solved by the same method employed by RMA2.

In numerical computations, the total water depth and the velocity components are first obtained by solving (1). The concentration of a pollutant (COD) is then predicted by solving (2).

## 3. Numerical Analyses

### 3.1. Application of Numerical Model to a Real Field

**Figure 2:**
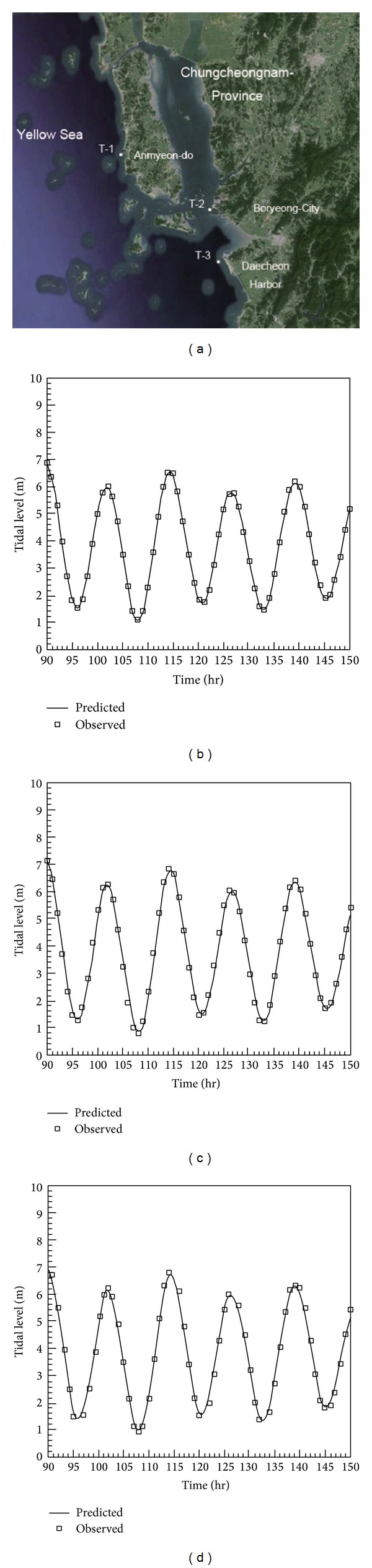
Application of the numerical model to a real field.

The numerical model employed in this study is first applied to predict variation of the tidal level in a real field and to test and validate the applicability of the model. The model [9] is used to predict the sea level near the Anmyeon-do Island located in the Yellow Sea (see Figure 2(a)). The sea is well known for a high tidal range (about 6.0 m). The tidal levels are measured at three gage stations as shown in Figures 2(b)–2(d). The measurements are taken for 60 hours. As shown in Figure 2, the predicted tidal levels agree very well with observed ones. Although the coastline near the Anmyeon-do Island is very complicated, the numerical model employed in this study provides reasonable results.

### 3.2. Computational Area

The area modeled in this study is located near the Youngil Bay in the southeastern region of the Korean Peninsula. The Hyeongsan River flows to the estuary adjacent to the Youngil Bay as shown in Figure 1.

The topographical map of the Hyeongsan River and Youngil Bay is obtained at a scale of 1/15,000 of a nautical chart. The length of the inland canal is 1.3 km, and the width designed is 20 m so that small cruise ships could sail both ways.

### 3.3. Model Conditions

For the computational domain, the turbulent exchange coefficient, and Manning's roughness coefficient *n* are taken equal to 2000 Ns/m^3^and 0.03, respectively. The turbulent exchange coefficient employed is from the previous study [10] and Manning's *n* value is obtained from a previous report [11]. Boundary conditions were determined by the ordinary water flow and sea level.

The finite element mesh describing the bathymetry of the modeled water body was constructed by using FastTABS. The number of quadratic mesh elements is 7,320 in the numerical model. The diffusion coefficients used for those areas were *D*_*x*_ = 0.1 m^2^/sec and *D*_*y*_ = 0.2 m^2^/sec [12].

**Figure 3:**
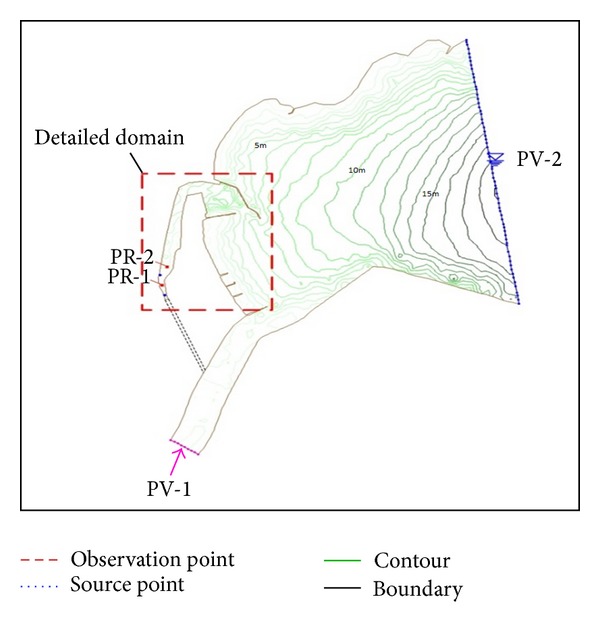
Topography of Dongbin Harbor and the Hyeongsan River.

A general layout of the computational domain is shown in Figure 3, in which PV-1 and PV-2 represent the upstream and downstream boundaries of the computational domain, respectively. The available COD concentration was 1.8 ppm measured on March 16, 2005, and it is employed as an initial condition along the upstream boundary in numerical computation. And the downstream boundary condition was considered as an open boundary. The numerical models of RMA2 and RMA4 were employed for the cases of before and after construction of the island canal. The width of an inland canal is fixed as 20 m.

## 4. Results of Numerical Computation

**Figure 4:**
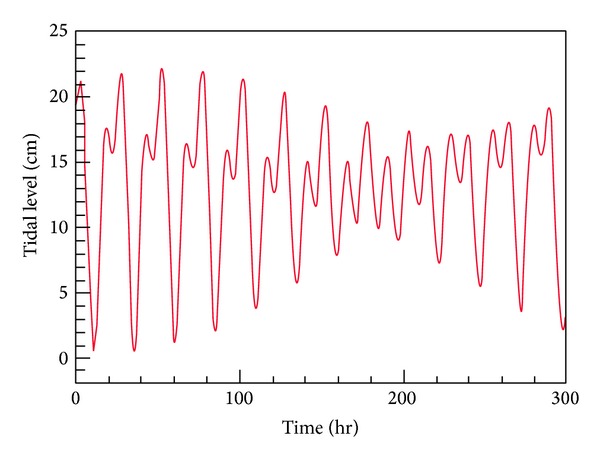
Predicted time history of tides at Youngil Bay.

The model is first used to predict the time history of tides at Youngil Bay.  Figure 4 displays the time history of tidal level at a station located inside the Youngil Bay. The maximum tidal range is about 0.22 m. While the tidal range is very large in the Yellow Sea, that of East Sea is relatively small. Thus, a construction of an inland canal is promising to improve the water quality inside the Dongbin Harbor by connecting with the Hyeongsan River.

**Figure 5:**
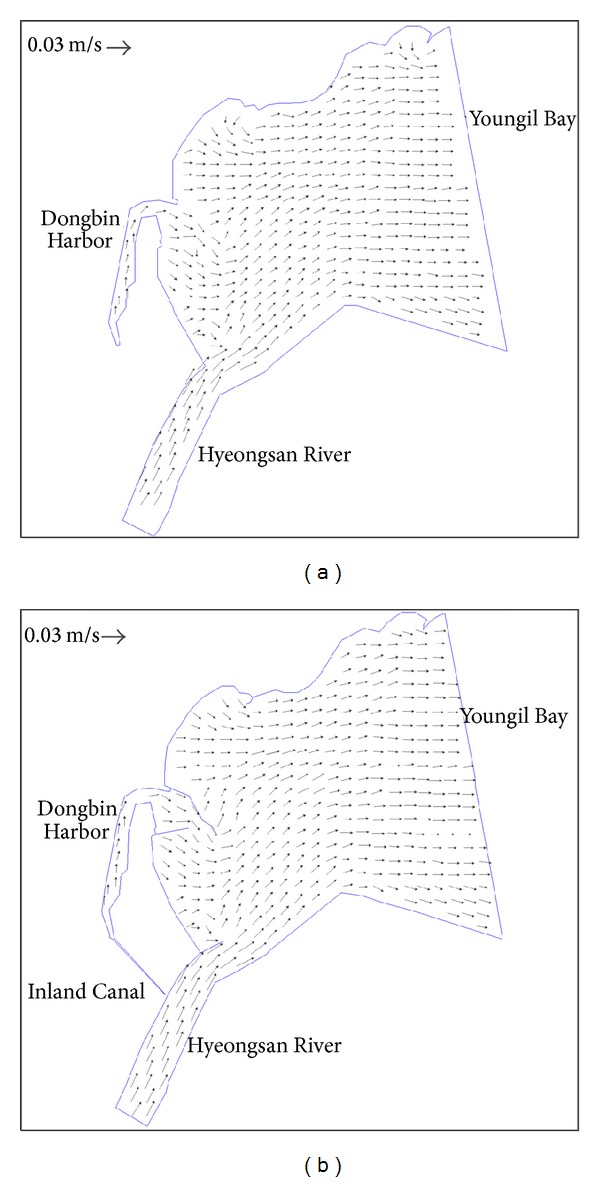
Velocity distributions before and after construction of an inland canal.

In Figure 5, a comparison is made among the cases of before and after construction of an inland canal in the area covering the Youngil Bay, Dongbin Harbor, the Hyeongsan River, and inland canal as marked in Figure 3. As shown in the figure, if observed closely the velocity distribution inside the Harbor is more active in Figure 5(b) after construction of the inland canal than Figure 5(a), which shows that the water body movement has increased after construction of canal. Although the distinction between two figures is obscure, if observed more closely, Figure 5(b) shows a relatively active velocity field than Figure 5(a) that shows the increased movement of water body in the canal. The predicted averaged velocity in the central region is about 0.058 cm/sec at the present in the Dongbin Harbor. On the other hand, the predicted averaged velocity is about 0.68 cm/sec for the inland canal, and the canal will increase the water supply from the Hyeongsan River to the Harbor. Thus, the water quality inside Harbor can be improved by constructing an inland canal.

**Figure 6:**
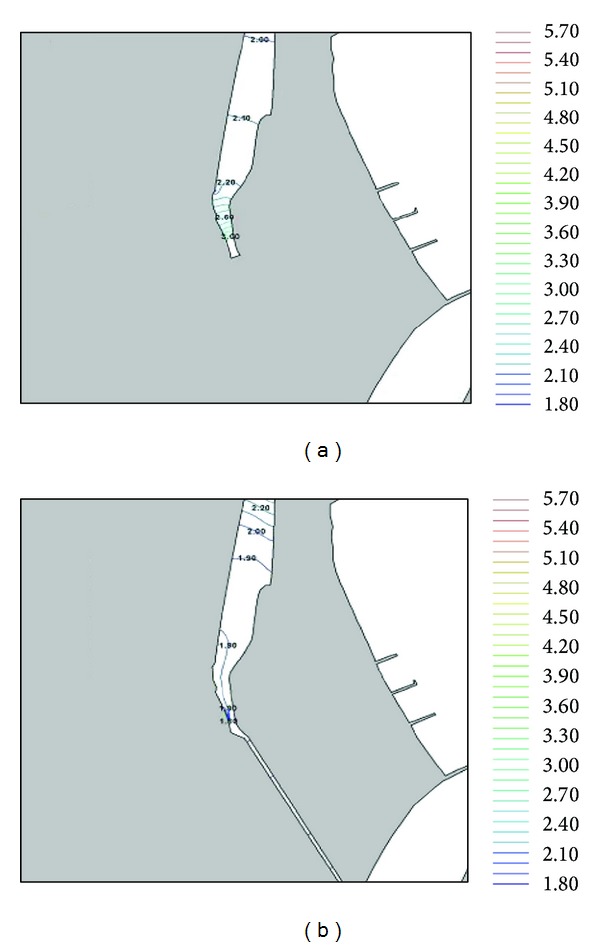
Concentration distributions after 30 days from the beginning of construction.

The COD (chemical oxygen demand) concentration is the decisive factor to assess water quality. Figures 6(a) and 6(b) and Table 1 show significant differences in concentrations before and after constructing the channel. The decrease in COD concentration shows that the water quality is improved.  Figure 6(a) shows the measured concentration distribution before construction of the canal, while Figure 6(b) shows the concentration distribution predicted by the RMA4 after construction of the canal.

In Table 1, the variation of COD concentration inside the Dongbin Harbor is displayed. A comparison is made between, before, and after construction of the canal. As shown in the figure and table, the water quality inside Dongbin Harbor is gradually improved. The measured COD concentration at PR1 is 3.00 ppm before the construction of an inland canal. However, the numerically predicted concentration is about 1.93 ppm after the construction. This shows that the water quality inside the Harbor is greatly improved.

### 4.1. Concluding Remarks

In this study, a series of numerical simulations were performed to determine the potential purification of polluted water inside the Dongbin Harbor by using RMA2 and RMA4. The flow characteristics inside the Harbor changed from the current stagnant flow to a flow which restored water quality after the construction of the canal.

After construction of an inland canal, stagnant water inside the Dongbin Harbor circulated out with the seawater flow. Both RMA2 and RMA4 simulated the cases of before and after construction of the inland canal. This study provides an effective design resource for construction of an inland canal.

## Figures and Tables

**Table 1:** Measured and predicted concentration of COD (ppm).

Before construction		After construction	
(Measured)		(Predicted)	
PR1	PR2	PR1	PR2
3.00	2.20	1.93	1.89
